# The Experience of European Researchers in China: A Comparative Capital Advantage Perspective

**DOI:** 10.1007/s13132-022-00982-3

**Published:** 2022-03-07

**Authors:** Andrea Braun Střelcová, Yuzhuo Cai, Wei Shen

**Affiliations:** 1 Max Planck Institute for the History of Science, Berlin, Germany; 2 Faculty of Management and Business, Tampere University, Tampere, Finland; 3 Deakin University, Melbourne, Australia; 4 EU-Asia Institute, ESSCA School of Management, Angers, France; 5 Zhejiang University, Hangzhou, China

**Keywords:** China, Europe, Academic migration, Research cooperation, Capital, Internationalization, Higher education

## Abstract

This paper unfolds a “black box” concerning European researchers working in China, an emerging phenomenon as an outcome of European and Chinese international research collaboration. China’s rapid economic growth has created plentiful career opportunities in research and innovation, attracting mainly returning Chinese but also non-Chinese researchers. While emerging research has brought attention to the individual experiences of the latter group, this work has been hampered by a lack of conceptual frameworks, as well as empirical knowledge regarding these migrants’ motivations, job satisfaction, and career prospects. This article attempts to bridge this research gap by focusing on European researchers working in Chinese universities. It develops a novel analytical framework that integrates insights from the push–pull framework and Bourdieu’s conceptualization of capital and applies this framework in a qualitative investigation of China-based European researchers. This analysis shows, first, that significant capital gains exist for natural scientists, as opposed to social scientists, in China, as the former operates in a more internationalized, better-funded, and less politically controlled environment. Second, it demonstrates that European researchers’ migration journeys in China remain predominantly temporary, as their initial advantages tend to fade away over time. Finally, this article finds that European researchers’ reasons for leaving China are connected to challenges beyond the workplace, yet their stay in China becomes an important step in increasing their competitiveness in the global academic labor market.

**Supplementary information:**

The online version contains supplementary material available at 10.1007/s13132-022-00982-3.

## Introduction

Many academics and scholars today have become globetrotters who move across the world in pursuit of science even more than other professions since international networking is a traditional norm in academia (Mahroum, 2000). The academic migrants also play an increasingly important role in the knowledge-based economy since they can create new ideas and innovations, thus leveraging economic growth (Zhang & Lucey, 2019). Academic migration as a particular form of human mobility entails various capacity mobility. Examining academic migration helps further understand the importance of human factors in innovation systems (Martinidis et al., 2021) from a transnational perspective. When the knowledge-based economy has evolved towards innovation ecosystems (Carayannis & Campbell, 2009) or Society 5.0 (Carayannis & Campbell, 2021), sustainable innovation/development profoundly depends on co-evolution and co-creation among innovation actors across not only sectors but also nations (Cai et al., 2020).

Against such a background, many countries are attempting to attract highly skilled migrants in a “war for talent” (Brown et al., 2008). The People’s Republic of China, which has suffered from “brain drain” to the West since reform and opening up in 1978, has long recognized the need to attract global talent for its national development (Zweig et al., 2008). China’s strategy to compete for global talent, however, has changed from encouraging overseas Chinese graduates to return (Shumilova & Cai, 2016) to employing returned Chinese scientists and even non-Chinese experts to contribute to the development of science, technology, and innovation in China (Yang, 2020). An increasing number of researchers from the West have moved to China or considered the option of working in China; it has been estimated that around 800–1000 European researchers are working full-time in China (EURAXESS China, 2021).

Such a phenomenon can be explained by three primary reasons. First, diminishing opportunities and increasing competition in Europe’s academic labor market have pushed many European researchers to seek more attractive career destinations elsewhere (Williams, 2016). Second, China appeals to some Western/European researchers, due to its rising leadership in science and technology (Basu et al., 2018), its status as the world’s largest source of scientific articles (Tollefson, 2018), and the second-highest level of spending on research and development (UNESCO, 2018), which result in further convergence of innovation gap between China and the European Union (EU) (Kowalski, 2020). Third, there has been growing interest among both the EU and China in science and innovation cooperation at multiple levels, such as governments, institutions, and individuals (Cai et al., 2019).

As the literature review presented later in this article indicates, although research on academic migrants to China has emerged, only limited scholarly literature exists on the experience and working environment of foreign researchers who live in China. For instance, the motivations, job satisfaction, and career prospects of Western researchers in Chinese higher education and research institutions remain largely under-researched. This gap also stems from a lack of appropriate analytical tools for understanding the phenomenon. Some studies did not apply analytical frameworks (e.g., Han, 2021) or were purely inductive (e.g., Larbi & Ashraf, 2020). Others mainly applied a push–pull model (Kuzhabekova & Lee, 2018) or Bourdieu’s capital conceptualization (Bauder, 2020). As further explained in the analytical framework section, either of these two analytical tools has its limitation.

This article, therefore, attempts to bridge research gaps in the literature on China-bound skilled migration by focusing on European researchers in Chinese academia. Particularly, we ask the following research questions: What motivates European researchers to pursue their careers in China? How satisfied have they been with their working conditions in China? What are their future career prospects? To address these research questions, we developed a novel analytical framework that integrates the insights from the push–pull framework, common for understanding academic mobility and migration, especially of students (e.g., Li & Bray, 2007), and Bourdieu’s conceptualization of capital (Bourdieu, 1984, 1986, 1990). Our primary research data are gathered from semi-structured interviews, conducted in 2017–2018 in China, with 28 China-based European researchers. In this article, we understand the phenomenon of European researchers working in China as academic migration, which is defined as a professionally motivated cross-border move intended to be permanent or beyond the length of one year (Bauder, 2020).

We chose to focus on the academic migration of Europeans to China because such researchers belong to the skilled migrants prioritized by the Chinese migration policies (Richter, 2020). European researchers are in demand in China due to their skills, educational background from global universities, and international networks—attributes that can contribute to China’s economic development (Center for China & Globalisation, 2017). Our findings suggest that European researchers’ migration journeys in China remain predominantly temporary, as their advantage and available opportunities tend to fade away over time. Their experience in China, however, becomes an important step in building their track records and increasing their competitiveness in the global academic labor market. The empirical analysis also demonstrates the usefulness of our constructed analytical framework—the framework of migration for comparative capital advantage.

To develop these ideas, this article starts with a review of existing research on the topic of migration to China, particularly academic migration, to identify research gaps. The following sections, then, introduce our analytical framework and research methods. From there, we provide a detailed presentation of our research findings that address the three research questions. Finally, we highlight both the scholarly and practical implications of our analysis.

## Literature Review

Historically, China has been a country of emigration, rather than immigration (Center for China & Globalisation, 2017), but since the turn of the twenty-first century, it has become a popular destination for a diverse set of migrants (Pieke, 2011). These changing flows are reflected in a small but growing literature on international migration into China (e.g., Farrer, 2019) that has primarily focused on immigrants from less developed countries who pursue jobs or trading activities outside China’s knowledge-intensive sectors (Haugen, 2012; Winders, 2014). A small volume of literature has also looked into skilled migrants who come to China to pursue better lives, including Western “expats” (Lehmann & Leonard, 2019).

The issue of foreign researchers migrating to new or emerging destinations such as China has been discussed, often indirectly, in five groups of literature: studies of skilled migration and related policies (e.g., Zweig & Wang, 2013), management research on academic expatriation and career development (e.g., Richardson & McKenna, 2002), higher education research on internationalization and mobility (e.g., Huang & Welch, 2021), China studies (Klotzbücher, 2014), and non-scholarly reports (e.g., Paramor & Shao, 2018). In the first group, studies have mostly focused on highly skilled migrants, including academics, and the related immigration and political framework (Liu & Ahl, 2018). Those exploring China-bound academic migration were, until recently, rare and tended to focus on these migrants’ experience as educators in Chinese academia (Kim, 2015), with a limited analysis of their experiences as scientists in the local research environment (Farrer, 2014).

The second literature group on expatriation and career development includes studies of international elites, including academic expatriates moving to Asia, such as Korea (Kim, 2016), Kazakhstan (Lee & Kuzhabekova, 2017), Turkey, Singapore, and the UAE (Richardson & Zikic, 2007) and highlights the relative lack of empirical knowledge regarding the expatriation of global academic talent, as opposed to skilled workers in the corporate sector, in terms of both motivations and cross-cultural adjustment experiences (e.g., Froese, 2012).

The third group features studies centered on foreign academics’ experiences and working environments in China, particularly those in officially approved Sino-foreign joint venture universities (Cai & Hall, 2016; Wang & Chen, 2020). However, the foreign academics discussed in these studies are not generally representative of Western academics in China, as Sino-foreign joint universities operate in a specific policy framework (Stanfield & Wang, 2012).

The fourth category includes analyses of the difficulties encountered by foreign (social) scientists when, for example, setting up collaborations or doing fieldwork in China (Heimer & Thøgersen, 2006; Klotzbücher, 2014). Although these studies were not conducted from the perspective of academic migration, their findings offer a glimpse of the research environment faced by Western academics in China.

The final set of non-academic literature, mainly journalistic accounts about foreign scientists in China, is quite developed and, to some extent, captures the reality of foreign scientists moving to China (e.g., Jia, 2018), including Europeans (Mervis, 2019). Some EU research and innovation policy projects in China also provide specific insights into European researchers’ experiences (EURAXESS China & Development Solutions, 2019).

Although the studies mentioned above do not directly address this article’s research subject, they shed useful light on our research in two aspects. First, some studies imply that the pull and push forces influencing foreign researchers in China can be seen from the perspective of symbolic capital (e.g., Kim, 2015) or social capital (e.g., Bauder, 2020). Thus, they inspire us to build our analytical framework of integrating the push–pull framework (e.g., Mazzarol & Soutar, 2002) with Bourdieu’s (1986) conceptualization of capital. Second, the literature mentioned above indicate both favorable conditions and potential challenges for foreign researchers working in China (Table 1). Although the discussion of these factors is quite general, it shows that the foreign researchers’ perception of push–pull factors may change with time. Particularly, certain push factors, this work demonstrates, were more likely to be perceived by academic migrants only after staying in China for a few years. The delayed push factors might affect migrants’ job satisfaction and eventually influence their career prospects.

**Table 1:** Favorable conditions and potential challenges for foreign researchers to migrate

**Factors pulling foreign researchers to China**	**Factors pushing foreign researchers away from China**
Opportunities for career growth Availability of suitable positions More research funding Better salary Desire to experience another culture Family reasons (e.g., spouse) Quality of life	Authoritarian political system and restricted freedom Significantly different culture and language Challenges securing a (long-term) visa or residency permit No significant salary increases No clear promotion framework

Source: Synthesized from Van Noorden (2012), Woolston (2020), and MORE3 (2017)

Nevertheless, none of the sources described above provide a systematic analysis of the conditions of academic migrants who choose to pursue a career in China. Existing empirical studies are also hampered by a lack of well-elaborated analytical tools. In particular, questions remain about foreign migrants’ decision to work in China, their job satisfaction, and their career prospects, with especially slim research on Europeans’ experience in Chinese academia.

A recent study by Han (2021), however, investigates international academics’ experiences in three Chinese universities (one traditional university and two Sino-foreign institutions) and is the closest research to this article. Han’s timely scholarly contribution reveals the experience of international academics in contextualized case settings, but only six of the 18 interviewees worked in the public university, and only two were from Europe (the UK). Moreover, Han’s findings were based on her own interpretations of the data, without the application of a solid analytical framework. This also justifies the need for developing a suitable analytical framework for understanding Western academic migration to China.

## Analytical Framework

To approach our research questions, we first construct an analytical framework that integrates insights from the push–pull framework in academic mobility and migration (e.g., Kuzhabekova & Lee, 2018) and Bourdieu’s conceptualization of capital (Bourdieu, 1984, 1986, 1990). While the push–pull framework and capital concept both have been applied in migration studies (Erel, 2010; Van Hear et al., 2017) as well as studies of academic mobility (Leung, 2013; Li & Bray, 2007), the synergy between the two can advance theoretical understandings of international academic migrants’ motivations, job satisfaction, and career prospects.

### Push–Pull Factors for Understanding Academic Migration

The classic push–pull model originally stressed the migrant’s economic cost–benefit calculation and later incorporated more sociological reasoning (Brettell & Hollifield, 2015). In higher education, the push–pull framework has mostly been applied to studies of international students (Mazzarol & Soutar, 2002), and international academic mobility and migration (Nunn & Price, 2005; Tremblay et al., 2014). The framework’s basic assumption is that an individual’s decision to migrate to a particular country in search of better opportunities is influenced by a combination of push–pull factors (Van Hear et al., 2017). Push factors relate to the sending country’s forces pushing migrants away (Lee, 1966). For instance, push factors in European countries are dependent on labor market conditions (unavailability of adequate job openings, lack of viable promotion options, disappointing work conditions) in combination with individual factors such as personal situation, age, or family status (Lee & Kuzhabekova, 2017). Pull factors refer to the elements attracting migrants into a receiving country (Ferwerda & Gest, 2020) and can be related to the prospect of better jobs or economic opportunities, or the country’s immigration policies aimed at attracting skilled migrants (Hayes & Pérez-Gañán, 2017; Van Der Wende, 2015).

Regardless of wide applications of the push–pull model in empirical migration studies in the context of higher education (e.g., Li & Bray, 2007) and beyond (e.g., Van Hear et al., 2017), the model has been criticized for being too static (Malmberg, 1997) and theoretically void (de Haas, 2011). This critique implies that a list of push–pull factors relating to the structural facets of sending and receiving countries (and the individual’s perception of those factors) is insufficient for providing theoretical explanations of the fluidity and complexity of the migration as a process. Therefore, we supplement the push–pull framework with Bourdieu’s conceptualization of capital (Bourdieu, 1984, 1986, 1990) to build a stronger theoretical approach to grasping the nature of academic migration.

### Bourdieu’s Conceptualization of Capital

According to Bourdieu (1984, 1986), capital can be understood as a currency that affects an individual’s position in the social world and can be divided into distinct, yet interconnected, forms: economic, social, cultural, and symbolic. Bourdieu’s capital is related to his theory of practice, especially the concepts of the *field* and *habitus*. The former is a social space in which interactions, transactions, and events occur (Bourdieu, 2005). The latter refers to individuals’ subconscious disposition which regulates their behavior (Bourdieu, 1977). In the process, individuals accumulate economic, cultural, social, and symbolic capital in order to gain a higher position within their field, with their actions being conditioned by a socially constructed habitus. Bourdieu’s capital theory has become a useful concept to explain academic mobility and migration (e.g., Al Ariss & Syed, 2011; Erel, 2010; Smith et al., 2019) for two reasons. First, the *field* concept provides a useful perspective to consider academia or higher education systems as a *global field* (Marginson, 2008; Mendoza et al., 2012). Second, the four forms of capital can be understood as resources through which academic migrants attempt to maximize their gains in the global field of academia (Mendoza et al., 2012). During the process, migrants develop new ways to leverage such resources to their advantage in the host society (Erel, 2010).

The first of the four forms of capital—economic capital—is the root form of all capital and refers to money and ownership of financial means, means of production, material goods, and other assets such as property (Bourdieu, 1986). The second form—social capital—can be understood as a network of contacts and the real or virtual sum of resources that could be mobilized through such networks (Bourdieu, 1986). The third form—cultural capital—can be divided into three subtypes: embodied, institutionalized, and objectified (Bourdieu, 1986). The embodied aspect of cultural capital is linked to the body and the mind; the institutionalized type takes the form of educational qualifications as a “certificate of cultural competence” (Bourdieu, 1986, p. 246), and the objectified aspect refers to material artifacts with cultural meaning. Finally, symbolic capital sits on top of each of the other three forms of capital, and derives its function from the social recognition, status or prestige, attached to certain competencies and values (Bourdieu, 1984), strongly relevant in the academic profession (Bourdieu, 1990; Mendoza et al., 2012). Table 2 provides a summary of examples of the four forms of capital from migration studies, as applied in academia.

**Table 2:** Forms of capital with some examples as applied to the academic labor market as a global field

**Forms of capital**			
**Economic**	**Social**	**Cultural**	**Symbolic**
Funding, grants	Scientific community	Language and other embodied skills	Publication track record
Research equipment	Association membership	Education background	Funding track record
Salary	Project consortia	Cross-cultural competences	Teaching, supervision experience
Awards, prizes	Research group/team	Books, research data, and other research-related artifacts	Reputation in the community

Source: Synthesis from Bauder (2020), Eddy (2006), Nahapiet and Ghoshal (1998) Leung (2013), Mendoza et al. (2012), and Grenfell (2008)

In contrast to the push–pull framework, which has been criticized for lacking a theoretical basis to hold together dispersed internal and external factors affecting individuals’ decisions (Castles, 2010), the capital perspective on migration helps “encompass micro-individual, meso-organizational, and macro-contextual influences that affect migrants’ career choices” (Al Ariss & Syed, 2011, p. 286). Despite the popularity of Bourdieu’s concepts in migration studies, scholarship in the field has not fully explored Bourdieu’s generative potential (Kim, 2018). While Bourdieu’s capital helps conceptualize the nature of academic migration, with the argument that “possession and utilization of capital are at the center of migrants’ success and the ability to thrive in their new environment” (Echa, 2018, p. 125), it has rarely been applied to highlight how sending and receiving countries create advantages or disadvantages for individual migrants’ capital (Kim, 2018). Such a comparative advantage perspective is more emphasized in the push–pull analysis.

While seeing the usefulness of Bourdieu’s capital concept in scrutinizing what advantage an academic migrant can take by moving from one job market to another, we are also aware of the concept’s possible limit in fully exploring such advantage. Bolin (2012) pointed out, “Bourdieu never really enters a serious discussion with Marx on value” (p. 39). Marx used the concept of valorization of capital to understand “the increase in the value of capital assets through the application of value-forming labor in production” (Hladchenko, 2016, p. 670). The issues concerning how capital can be re-invested were not much discussed by Bourdieu (Bolin, 2012). Our referring to the literature comparing Bourdieu’s and Marx’ capital theories is not to join a Marx-Bourdieu debate, which is beyond this article’s focus. Rather, our purpose is to borrow the insights of Marx’s concept of valorization concerning the value increase of capitals when building our analytical framework. Specifically, we define valorization of capital in our research as a process of making capital, previously acquired by an academic migrant, increase in amount, volume, or value, over time, in the receiving country.

### Migration for Comparative Capital Advantage: Integrating Push–Pull Framework and Bourdieu’s Conceptualization of Capital

A robust comparative perspective in the push–pull framework inspires us to strengthen the use of Bourdieu’s capital concept for comparing the valuation of capital in the sending country against its valuation in the potential receiving country. Comparing migrants’ position in the sending country with their gains in the receiving country is a standard micro-theoretical approach in migration studies (Massey et al., 1993). The novelty of our framework of comparative capital advantage lies in the following three aspects. First, we include all four capitals according to Bourdieu’s original conceptualization into our comparative lens. So far, existing approaches comparing migrants’ gains and losses have rarely systematically applied Bourdieu’s capital. Instead, they have taken a relatively narrow focus on some of the capitals, such as social capital (e.g., Bauder, 2020) and cultural capital (e.g., Erel, 2010).

Second, when comparing capital advantages, we look at two dimensions of valuation of capital in the sending country against its valuation in the receiving country: (1) gaining new capital through a work position in a new environment and (2) valorizing existing, previously acquired, capital. The comparison of capital valuation between sending and receiving countries is the core of a migrant’s assessment of the push from the sending country and the pull from receiving country for making migration decisions. Thus, our comparative analysis involves both structure (macro) and migrant’s agency (micro). Similar approaches in existing research mainly examine new capital gained through migration (Bauder, 2020) without considering potential value changes of existing capital. Some rare studies have considered how migrants find new ways and creative mechanisms to use their existing capital (Erel, 2010; Kim, 2018) but have not linked this dimension into the analysis of migrants’ actions in shaping their comparative advantage between sending and receiving countries.

Third, the comparative capital advantage highlights a global perspective, beyond the dichotomy of two countries, one sending and one receiving. We see migration (between Europe and China) taking place in a global field of academia, characterized by the “globalization of positional competition” (Brown, 2000) or, particularly, increasingly intensified global competition in the field of higher education (Marginson, 2008). Such a global perspective enables a deeper understanding of academic migrants’ career trajectories, in that working in a foreign country (e.g., China) is not the end of the academics’ career goals but part of their efforts to maximize their positions in the global positional competition. Current migration studies have begun to pay more attention to the global comparative context (Winders, 2014), but the call for research on changing global, multifaceted, and diverse migration flows continues. In the absence of a general theory of migration and reliable statistical data (Castles, 2010), there are no well-elaborated theoretical or analytical tools for the study of global migration that analyzes the comparative advantage between sending and receiving countries from a global competition perspective. However, our framework accounts for both individual micro-perceptions and macro-processes as it sets the national characteristics in light of the global transformations.

**Fig. 1:**
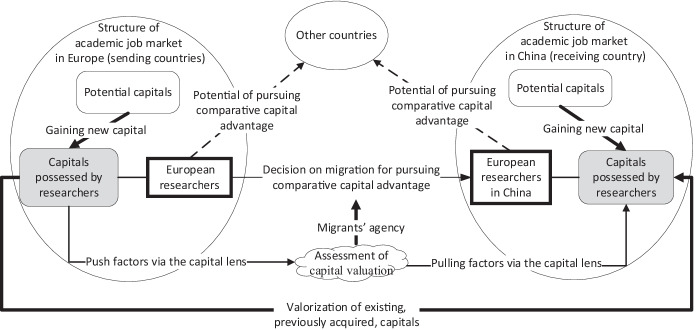
The analytical framework of migration for comparative capital advantage

Following the three novel perspectives mentioned above, we construct the analytical framework of migration for comparative capital advantage (Fig. 1). There are three core elements in the framework: European researchers in China, their new capital gains in China, and the valorization of their capitals acquired in Europe when migrating to China. The three elements must be observed in broad contexts, including the academic job markets in Europe and China and the wider global field of academia.

Our focus of analysis is on how academic migrants compare the chances of gaining and valorizing capitals, in the receiving country (i.e., China) with those in their sending countries in Europe (Table 3). In the empirical analysis, we specifically identify two patterns: first, how European researchers perceive their comparative capital advantage/disadvantage between Europe and China and, second, how those advantages/disadvantages are reflected in their gaining or valorizing different forms of capital. The underlying assumption is that European researchers’ decisions to migrate and remain in China are driven by their perceived comparative capital advantage.

**Table 3:** Framework for analyzing the motivations, job satisfaction, and career prospects of European researchers working in China

**Research** **inquiry** **Type of capital**	**Motivation before moving to China (past)**	**Job satisfaction (present)**	**Career prospects** **(future)**
**Economic capital**	How do European researchers perceive their potential comparative capital advantages (or disadvantages) of working in China?	How are the expected comparative capital advantages/disadvantages (before migration) compared with perceived capital advantages (or disadvantages) of working in China (after migration)?	How do China-based European researchers foresee their comparative capital advantages (or disadvantages) of returning to Western countries?
**Social capital**			
**Cultural capital**			
**Symbolic capital**			

## Methodology

As this article investigates an understudied topic, we apply an explorative qualitative research method that is suitable for gaining a deeper understanding of lived experience (Creswell, 2014). Nevertheless, the research was not purely inductive, as is typical in a grounded theory approach (Strauss & Corbin, 1990). Rather, the analysis was guided by an analytical framework, as “the use of theory… not only is an immense aid in defining the appropriate research design and data collection but also becomes the main vehicle for generalizing the results of the … study.” (Yin, 1994, p. 32).

The qualitative data mainly come from in-depth semi-structured interviews conducted with 28 European researchers in China in October–November 2017 and April–May 2018. Interviews took between 20 min and over 2 h, with an average length of about one hour. Respondents were identified through direct professional contacts and snowball sampling to maximize relevant interviewees, due to the specificity of our target group and to strong connections among people in this group. Thus, it was necessary to ask interviewees to recommend potential participants from their circles to get leads for other interviews.

Detailed descriptions of the interviewees’ profiles are included in the Online Appendix. We attempted to ensure diversity among interviewees in terms of the following attributes: gender (10 female, 18 male), academic career stage (21 mid-career or senior researchers, 1 postdoctoral researcher, and 6 doctoral students, all but one in late stages of their PhD), academic field (14 natural and life sciences, 14 social sciences and humanities), the priority of research area (15 high, 13 low, according to whether the field was listed as a priority in China’s National Medium & Long-Term Plan for Science & Technology, the National Natural Science Foundation of China, or the Ministry of Science and Technology’s Key Programmes), length of stay (17 interviewees had spent more than 5 years at the time of the interview in China, 13 less than 5 years), resident city (11 worked in Beijing, 13 in Shanghai, 4 in other cities), whether they looked after children (13 did not, 8 did, and 7 did not provide this information), and institution (20 were based at one of the 39 elite institutions from Project 985, 4 at one of the 112 key higher education institutions listed in Project 211, and 4 at a research institute outside the university system). Interviewees were working at a total of 16 public Chinese universities and research institutes. Among them, 27 were ethnically Caucasian, and one was of mixed Chinese-European heritage. We included several doctoral students since they formally classify as early career researchers under the EU researchers’ career framework (EURAXESS, 2020).

Interviews, conducted and transcribed in English, were analyzed by using three coding strategies. First, interviewees’ profiles were coded, using the attributes mentioned above. Second, the analytical framework (Table 2) was applied to code interview transcripts and provide direct answers to the research questions. Third, open coding (through inductive reasoning) was used to identify possible new patterns within or outside the analytical framework.

It is important to note that the interviewer (Andrea Braun Střelcová), a European living in China when the interviews were conducted, had been a long-term resident there and a part of the foreign researchers’ communities, although not a researcher herself. Interviewees may have been more open to an interviewer familiar with their situation, which was a unique advantage in the attempt to mitigate potential challenges in data collection common for researchers in authoritarian political regimes (Glasius et al., 2018). Moreover, given the lack of statistics and information about European researchers in China, the interviewer had the best knowledge about how to locate interviewees who represented diverse profiles. To avoid the interviewer’s biased interpretation of the data due to her proximity to interviewees, we tried to use an analytical framework to structure the empirical analysis and utilize the second author, who was not involved in the community, to question and critically reflect on the interview analysis. Nevertheless, some level of subjectivity is likely to remain in the data analysis, which is a general characteristic of qualitative research (e.g., Silverman, 2010).

## Research Findings

This section presents our answers to the three research questions concerning European researchers’ motivations, job satisfaction, and career prospects in China, based on our data analysis. Besides identifying overall tendencies, we focus on discovering variations within each pattern and show that the most significant contrasts in the experience of European researchers in China were between research fields—namely, social sciences and humanities (SSH) and engineering and natural scientists (ENS)—and, to some extent, between career stages. When it came to the consideration of leaving China, those with dependent children were more concerned with social factors. Although the literature on gender and academic mobility suggests that it is harder for female researchers to be internationally mobile (Jöns, 2011), in our analysis, we did not find evidence for greater satisfaction of male over female migrants. Furthermore, there were no significant differences in other attributes, such as the type of institutions, place of residence and nationality.

### Motivations: Expected Comparative Capital Advantage of Working in China Before Migration

Interviewees reported a prevalent dissatisfaction with the working and living conditions in their sending countries. Before the move, interviewees were based in Europe (*n* = 21) or the USA (*n* = 7). Some wanted to advance to the next career level but could not find an adequate faculty position (*n* = 9) or had professional success but were simply not satisfied with conditions at home (*n* = 4). For instance, interviewee 14 said, “I was a funding cash cow for my institution. I was successful at what I was doing, gaining lots of industrial funding but had no space to be independent and develop my own research.”

Regardless of the negative views about their situation, interviewees did not find planning to work in China to be an easy choice and described being initially “skeptical” about the move (*n* = 6). They further described their feelings as “scary” (interviewee 28) or “risky” (interviewee 12) or voiced a need for a “trial period” (*n* = 3). Several interviewees implied that they took a full-time position in China only because they could not find a suitable job in the West (*n* = 6), meaning that they accepted the job offer because of an expected comparative capital advantage in China, compared to the possible lack of viable options in their sending countries. The specific advantages for migrants in terms of both gaining new capital and valorizing existing capital are summarized in Table 4.

**Table 4:** Motivations of European researchers to move to China

**Forms of capital**	**Type of capital valuation**	**Expected benefits of potentially working in China in comparison with working in Europe (comparative capital advantages)**	**Mentioned by**
**Economic capital**	Capital gaining	Because of historically unprecedented growth with significant investments in higher education, research, and innovation in China, there would be opportunities, interesting job offers, or research funding, which are hard to find elsewhere	ENS: 13 SSH: 15
	Capital gaining	Some Chinese institutions can offer more competitive packages	ENS: 10 SSH: 4
	Capital gaining	Dual career offer for both partners	ENS: 3 SSH: 1
**Social capital**	Capital gaining	Desire to grow a network with Chinese scientists	ENS: 2
	Capital valorization	Already been invited to work in China through existing contacts, e.g., collaborative partners, friends, former supervisors, former students, and old colleagues	ENS: 7 SSH: 2
	Capital valorization	An offer based on existing inter-institutional cooperation	ENS 2 SSH 1
**Cultural capital**	Capital gaining	There would be unique opportunities to access region-specific data, e.g., fieldwork, archives, or other materials	ENS: 3 SSH 10
	Capital valorization	Early experience of travelling, studying, or volunteering activities in China sparked an interest	ENS 1 SSH 7
**Symbolic capital**	Capital gaining	Chinese universities can offer jobs in their pursuit of attracting researchers with a high track record potential	ENS 2 SSH 2

As Table 4 indicates, European researchers, regardless of research fields, expected comparative capital advantages in the form of economic and symbolic capital. Although the salary in China could not match North American or Western European standards, together with the additional benefits (e.g., housing subsidies, start-up funds, or a dual job offer for both partners), working conditions were attractive. Sometimes, economic capital (e.g., having a competitive offer) and symbolic capital (e.g., their research record being appreciated in China) were closely interrelated. As noted by interviewee 12,

I was interested in Asia, I went to Japan a few times, and I saw that Chinese science was improving a lot. It became obvious to work with them. To be honest, my CV was not good enough to go to MIT or Caltech, but in China, they were really interested. When I got the offer, I did some brainstorming with my family. First, they said: Are you crazy? But in the end, we decided to take the chance.

The expected comparative capital advantage in the form of social and cultural capital differed between SSH and ENS researchers. The data suggest that ENS researchers often came to China through a job offer or invitation through existing contacts (valorizing their previously acquired social capital), sometimes in combination with family reasons (e.g., a Chinese spouse). ENS researchers, in other words, migrated to China to pursue research without previous cultural capital such as knowledge of the Chinese language or significant experience in China through previous stays. In contrast, SSH researchers were split in two groups: those who came for a specific opportunity and were more similar to ENS researchers (*n* = 7) and those who were motivated primarily by China itself (*n* = 7). Both were interested in gaining or valorizing country-specific cultural capital (*n* = 10).

### Job Satisfaction: Expectation Vs. Reality

This section compares interviewees’ expected comparative capital advantage before moving to China with the reality after moving to China. The post-arrival initial period was often complicated by lack of knowledge of the local system (*n* = 6), a tedious Chinese bureaucracy (*n* = 6), and high pressure for delivering publications (*n* = 3). Nevertheless, most interviewees’ job satisfaction level was high, as they experienced a steep learning curve in the launch of a new life chapter. We distinguish among three kinds of experience about the working life in China: (1) Reality exceeded expectations; (2) Reality met expectations; and (3) Expectations were not met (summarized in Table 5). The first two were considered satisfying experiences, and the last was considered unsatisfactory.

**Table 5:** Job satisfaction of European researchers in China

**Expected capital advantage vs capital advantage in reality**		**Characteristics of the research participants**
**Satisfied**	**Reality exceeded expectations in the following aspects:** • Successful funding applications (gaining economic capital) • Access to infrastructure and facilities (gaining economic capital) • Work in more international fields (gaining social capital) • Institutional and team support (gaining social capital) • Talented students (gaining social capital) • Increases in local professional network (gaining social capital) • Access to a new culture and its specific research data (gaining cultural capital) • Freedom to carry out research (potential of gaining symbolic capital)	*n* = 9 Attributes (*n*): *Category*: Basic (7), applied (2) *Field*: ENS (9) *Priority*: High (9) *Seniority*: Senior (9) *City*: Beijing/Shanghai (6), other (3) *Institution*: 985 universities (3), 211 universities (2), research institute (4) *Gender*: Male (7), female (2) *Children*: Yes (3); no (4), N/A (2) *Length of stay*: < 5 years (3), ≥ 5 years (6)
	**Reality met expectations:** • Sufficient funding (gaining economic capital) • Previous experience in China helped to assess the situation before the migration (valorizing existing cultural capital) • Appreciation of previous work experience and educational credentials by employers (valorizing existing cultural capital) • Willingness to adapt and understand the system from the local perspective (gaining new cultural capital) • Environment perceived as opaque (disadvantage in gaining social capital) • Language barriers (disadvantage in gaining cultural and social capital) •The challenging yet steep learning curve of life in a new country (challenges in gaining cultural capital)	*n* = 13 Attributes (*n*): *Category*: basic (5), applied (8) *Field*: ENS (4), SSH (9) *Priority*: high (7), low (6) *Seniority*: junior (3), senior (10) *City*: Beijing/Shanghai (12), other (1) *Institution*: 985 university (11), 211 university (2) *Gender*: male (8), female (5) *Children*: yes (5); no (2); N/A (6) *Length of stay*: < 5 years (4), ≥ 5 (9)
**Unsatisfied**	**Expectations were not met:** • Less funding (disadvantage in gaining economic capital) • The realization of the complexity of work in an isolated academic system (disadvantage in gaining social capital) • Work in less internationalized fields (disadvantage in gaining social capital) • Separate research culture requires adaptation beyond what is feasible (disadvantage in valorizing cultural capital)	*n* = 6 Attributes (*n*) *Category*: SSH (6) *Field*: basic (3), applied (3) *Priority*: low (6) *Seniority*: junior (4), senior (2) *City*: Beijing, Shanghai (6) *Institution*: 985 (6) *Gender*: male (3), female (3) *Children*: no (6) *Length of stay:* < 5 years (3), ≥ 5 (3)

1. Reality exceeded expectations
  All interviewees in this group were senior ENS researchers. Despite initial skepticism about moving to China, their experience in the workplace surpassed their expectations. The gains in economic, social, and symbolic capital were bigger than they had expected. After they adjusted to life in China, its dynamic environment enabled them to apply for funding, access top-notch facilities and modern research infrastructures, materialize ideas quickly, launch projects, hire students, and carry out research. This environment led to a boosted track record in publications and research funding. Their rapid growth in career development also benefitted from significant research freedom as they observed looser regulations regarding ethical reviews, data protection, or animal experiments (*n* = 8). Moreover, they gained access to social capital in the form of a network among Chinese students and researchers, although less than foreseen due to language barriers (*n* = 3). These capital gains were described by interviewee 28:
  China is rising; there is funding. All the calls I applied for, where I could, I won the grant. I was a postdoc in Europe, now in my CV, I can show projects where I am a PI or a partner PI, and in most of the cases, I thank China for that. I am invited to more international calls to participate, and I always emphasize my network in China. Had I stayed in Europe, I would never be able to improve my CV so much.
  Comparatively, interviewees in this group experienced less progress in terms of gaining new cultural capital, but none felt professionally limited because they could not speak fluent Chinese. Although most funding applications were submitted in Chinese, they could get support from team members, colleagues, and services provided at the organizational level. Therefore, these researchers relied on assistants or colleagues to translate and provide informal advice on funding applications (and, once awarded, budget management). Interviewee 1, for example, noted that the translation and supporting services “show that transfer of knowledge is happening. Otherwise, you could just run your lab and be disconnected.”
2. Reality met expectations
  The second group (*n* = 13) was the most heterogeneous in terms of disciplinary areas, research fields, employing organizations, and family status. What they had in common was a preparedness to acquire knowledge about the working situation in China before migration. Before migrating to China, they frequently visited China and engaged in cooperation with local institutions (*n* = 8). Interviewee 16 shared her views:
  If you’re a strong, well-experienced international, you can do it. If you’re a European coming here with European ideals, it is hard. You need to have done some substantial experience or work outside of Europe to survive here well. It is not about visiting, but it’s about living, and it’s not about surviving, but it’s about thriving, and in order to do that, you need a different outlook. Things will not work the way they work in Europe. You need to adapt and observe.
  People in this group were content with the availability of research funding (*n* = 12) and support from their institution (*n* = 8). They also displayed a willingness to adapt to and understand the system from a local perspective (*n* = 9) to be better embedded in the local research community. Nevertheless, they still perceived the local environment as opaque, with frequently changing regulations and burdensome bureaucracy (*n* = 10).
3. Expectations were not met
  The third group was homogeneous, as they were all SSH researchers (*n* = 6), with most at a junior career stage at the time of the interview (*n* = 4). Although all had been highly motivated to come to China, they were disappointed after arrival. As interviewee 19 pointed out,
  When I arrived, things were not up to my expectations. The topic I wanted to do was probably too political, and the supervisors didn’t want to take the risk and responsibility. But they don’t tell you, “You can’t do it,” instead, they are trying to nudge you in a different direction. What my supervisor wanted me to do was not what I wanted to do. It was disappointing, and I didn’t feel connected to the research I was supposed to be doing.

New social and cultural capital was difficult to gain, due to differences in research culture, as well as financial, administrative, and governmental barriers (*n* = 6). Unsatisfied researchers experienced professional isolation that hindered their research plans (*n* = 5) and over time led to not only limited research results but also physical and mental health risks (*n* = 2). Some reported insufficient local funding (*n* = 3) and difficulties in acquiring data and accessing fieldwork opportunities (*n* = 4).

The challenges faced by these SSH researchers also seem to be discipline related. Due to the nature of their research, SSH researchers relied on access to local data through libraries, archives, or fieldwork. However, since most SSH researchers worked in fields not considered a high priority in China and much less international, interviewees perceived local funding as low or not available to them and their own income as low in comparison with international standards.

### Future Career Prospects: Decreasing Job Satisfaction and Seeking New Comparative Capital Advantage Elsewhere

#### Decreasing Job Satisfaction

Over time, the room to gain new capital tended to fade for European researchers in China. Researchers became more aware of their position within the system and believed they had exhausted their opportunities. Although reflections on specific capital gains differed between ENS and SSH researchers, the overall tendency was that job satisfaction declined after the early stage (Table 6). In other words, after working in China for several years, interviewees perceived that they were losing their comparative capital advantage.

**Table 6:** Losing comparative capital advantages

**Form of capital**	**ENS/SSH**	**Examples of losing comparative capital advantages**	**Attributes**
**Economic**	ENS	Large funding hard to get due to explicit or implicit ethnicity or citizenship requirements Glass ceiling, unclear promotion framework	*n* = 5 *Category*: Basic (3), applied (2) *Priority*: High (5) *Seniority*: Senior (5) *City*: Beijing/Shanghai (5) *Institution*: 985 university (5) *Gender*: Male (5) *Children*: Yes (3), no (2) *Length of stay*: ≥ 5 years (5)
	SSH	Income not high enough to cover all expenses Few funding sources for foreign researchers Access to equipment, research infrastructure not as good	n = 9 *Category*: Basic (5), applied (4) *Priority*: Low (8), high (1) *Seniority*: Senior (3), junior (6) *City*: Beijing/Shanghai (9) *Institution*: 985 universities (8), 211 (1) *Gender*: Male (6), female (3) *Children*: Yes (2), no (7) *Length of stay*: ≥ 5 years (5), ≤ 5 (4)
**Social**	ENS	Difficult integration in the workplace Gradual loss of international visibility Difficult to travel internationally or take sabbaticals	n = 8 *Category*: Basic (4), applied (4) *Priority*: High (8) *Seniority*: Senior (8) *City*: Beijing/Shanghai (7), other (1) *Institution*: 985 universities (5), 211 (2), RI (1) *Gender*: Male (7), female (1) *Children*: Yes (4), no (3), N/A (1) *Length of stay*: ≥ 5 years (4), ≤ 5 (4)
	SSH	Lack of information on how to access jobs or funding Tighter ideological control leads to professional isolation Cooperation hard to set up	*n* = 7 *Category*: Basic (3), applied (4) *Priority*: Low (7) *Seniority*: Senior (2), junior (5) *City*: Beijing/Shanghai (7) *Institution*: 985 universities (6), 211 (1) *Gender*: Male (4), female (3) *Children*: Yes (2), no (5) *Length of stay*: ≥ 5 years (6), ≤ 5 (1)
**Cultural**	ENS	Difficult to adapt to the local institutional culture Exclusion from some opportunities due to ethnicity Difficult to learn the language up to a point of professional fluency while having a full-time job Tricky to order material from abroad or exchange research data	*n* = 7 *Category*: Basic (5), applied (2) *Priority*: High (6) low (1) *Seniority*: Senior (7) City: Beijing/Shanghai (6), other (1) *Institution*: 985 universities (4), 211 (1), RI (2) *Gender*: Male (5), female (2) *Children*: Yes (3), no (3), N/A (1) *Length of stay*: ≥ 5 years (4), ≤ 5 (3)
	SSH	Understand but cannot adapt Restrictions on fieldwork, data collection Increasingly strict ideological control	*n* = 6 *Category*: Basic (3), applied (3) *Priority*: High (2), low (4) *Seniority*: Senior (2), junior (4) *City*: Beijing/Shanghai (6) *Institution*: 985 universities (6) *Gender*: Male (3), female (3) *Children*: Yes (3), no (3) *Length of stay:* ≥ 5 years (4), ≤ 5 (2)
**Symbolic**	ENS	Reputation as a “guest,” visitor, or bystander, rather than a colleague No clear promotion framework—no growth in professional power Exhausted track record growth potential Fearing loss of global visibility may affect reputation	*n* = 7 *Category*: Basic (4), applied (3) *Priority*: High (7) *Seniority*: Senior (7) City: Beijing/Shanghai (6), other (1) *Institution*: 985 universities (4), 211 (1), RI (2) *Gender*: Male (6), female (1) *Children*: Yes (3), no (4) *Length of stay:* ≥ 5 years (4), ≤ 5 (3)
	SSH	Questions added value as a foreign employee in SSH in China No clear promotion framework—no growth in professional power Professional isolation	*n* = 9 *Category*: Basic (5), applied (4) *Priority*: Low (9) *Seniority*: Senior (6), junior (3) *City*: Beijing/Shanghai (9) *Institution*: 985 universities (8), 211 (1) *Gender*: Male (4), female (5) *Children*: Yes (2), no (5), N/A (2) *Length of stay*: ≥ 5 years (7), ≤ 5 (2)

Although a variety of issues were related to losing comparative capital advantages (Table 6), most were connected to or directly caused by China’s academic and overall social environment, with which interviewees became familiar after several years of residence. Academic freedom was considered the biggest concern by SSH interviewees, who cited shrinking space for independent research manifested through the growing need to adapt to the usage of “approved” cartographic materials or “correct” historical narratives in the classroom and in their writing (*n* = 7). Interviewee 8 summarized the issue with a metaphor of “two TV channels” that shows the differences between the Chinese and Western academic systems in terms of social norms and regulations:

The two systems are like two different TV channels. You have to constantly switch between them. In the West, we like to publish articles that engage critically with China, but China has a different analytical vision; they rather praise China and analyze foreign things for their use-value, to see how these can help China. China has a large pool of local talent, and in social sciences and humanities, a foreigner can be a liability. What is our added value?

Some ENS researchers also pointed to a clear trend of intensifying ideological control over universities and research institutes and mentioned the call from their employers to double-check maps (*n* = 1), the need to align their research with political priorities, such as the Belt and Road Initiative (*n* = 3), or fieldwork restrictions (*n* = 1). Moreover, when working in Chinese public universities where foreign staff were rare, European researchers could face professional isolation. As interviewee 20 described,

In my department, I am the only non-Chinese among eighty. There are many things going on that I cannot master. It took me energy to start interesting projects, I was isolated, I did everything by myself. I was an alien in the department.

The broader social environment was mainly detrimental in three aspects. First, the legal framework and strict conditions for residency were obstacles for most interviewees (*n* = 23). Conditions for long-term residency were legally difficult to fulfil. The visa and work permit applications required lengthy renewal periods, even though interviewees had high educational qualifications and were, therefore, supposed to be in high demand by China. For example, only one interviewee had a permanent residency status with a Green Card, and only one interviewee was accurately informed about how to claim retirement or social benefits in the Chinese system. Most Europeans in China probably did not count on retiring there anyway as they perceived it too difficult to stay until retirement (*n* = 25).

Second, overall integration into local society proved challenging because of cultural barriers. Most interviewees (*n* = 24) were based in Beijing or Shanghai, big cities with a vibrant international community. Some foreigners were relatively well integrated, but others did not see the need to integrate, citing the fact that foreigners’ long-term presence was a relatively new phenomenon in a rapidly evolving Chinese society. Some complained of living in a “bubble.” Their inner struggles can be summarized by interviewee 1:

Self-relegated to a beautiful corner, a cage, I live in a bubble with other foreigners. This can be good for work, and it can be entertaining at the beginning, but after a while, you want to come back to life, to reality.

Third, the economic pressure of living in China forced research participants to consider relocating again. These pressures included high living expenses, especially in Beijing and Shanghai, and the sky-high costs for international insurance, healthcare, and schooling for children. Notably, foreigners considered it impossible to send their children to local public schools unless one parent was a Chinese citizen. Partners on a spouse visa could not legally work, and a single salary was too low to afford expensive international schooling. In addition, some reported air pollution, food safety, environmental degradation, perceived lack of aesthetic beauty in Chinese megacities, and feelings of discomfort of living in a commercialized, competitive society where ethnic or gender discrimination was often the norm (*n* = 10).

#### Seeking New Comparative Capital Advantage Elsewhere

When China-based European researchers realized that there would be a limited future for them there, they started looking for other opportunities or places where they could regain their capital advantage. Several interviewees expected that decreasing job satisfaction in China would ultimately lead to reverse migration, as international employees would leave China. As demonstrated by interviewee 12,

I was very successful, and I am very happy, but I think I was successful too early. I have already used all available funding programmes while I should have waited. Now the years are becoming critical, which money to apply for and how if I want to stay long-term? I don’t know what to get in the future. I also have my family, and life costs a lot.

As such, these European researchers in China were likely to be attracted by openings available on the global academic job market. Many expressed their desire to leave, although they strongly appreciated the experience in China (*n* = 14), and some had already secured a position abroad or were negotiating one (*n* = 6). Interviewee 8, who had at the time of interview just accepted a full-time job offer in Europe, said,

In terms of career prospects, Europe is a much more reliable choice. What I learned in China will be more appreciated in Europe than in China. China is a separate academic system in terms of the job market, far more unpredictable and difficult to navigate. As a foreigner, you might not have all information, you are missing out if no one ushers you in. Then it is the visa, the personal life quality, and ability to plan my future. The story might have been different if I had a Chinese spouse, but most people, even though they have a great time, in the long run, want to get away.

## Conclusion

This article investigated the motivations, job satisfaction, and career prospects of European researchers in China. In so doing, it constructed a novel analytical framework by integrating insights from the push–pull framework and Bourdieu’s conceptualization of capital. Through the analytical lens, it, for the first time, provided the most comprehensive and nuanced analysis of China-based European researchers’ experience.

Specifically, our analysis reveals the following findings. Regarding European researchers’ *motivations* to work in China, we found that European researchers in China were driven by their expected comparative advantage of both gaining new economic and symbolic capital and valorizing their existing capital, such as social and cultural capital. However, ENS and SSH researchers differ in their expectations on social and cultural capital dimensions, respectively. Regarding *job satisfaction*, most European researchers were satisfied with their job in China, although they faced adjustment challenges. However, there are variations between senior ENS researchers and SSH researchers. The former enjoyed more comparative capital advantages (in all four forms of capital) than they had expected before arrival. Their knowledge about the Chinese working environment gleaned from previous visits to China, as a China-specific cultural capital, helped mitigate risk and manage expectations before arrival. SSH researchers, however, were disadvantaged in gaining economic and social capitals in China. They faced professional isolation detrimental to their careers, especially if they lacked sufficient academic experience, professional networks, and a research track record. Regarding the future *career prospects*, the longer European researchers worked in China, the better they were able to see the challenges related to Chinese society and academia, which negatively affected their job satisfaction level. Therefore, European researchers were likely to utilize the capital they accumulated in China to advance their careers elsewhere in the long run.

Although our research focuses on academic migration, it also contributes to studies on knowledge-based society or innovation ecosystems. Specifically, it enhances theoretical understandings of academic migration, as an essential phenomenon in innovation ecosystems that consist of “fractal, multi-level, multi-modal, multi-nodal, and multi-lateral configurations of dynamic tangible and intangible assets” (Carayannis et al., 2018, p. 148). Migrant academics, associated with both tangible and intangible assets, are key actors in promoting/facilitating international research and innovation cooperation in multi-modal and multi-nodal systems. Our conceptualization of academic migration from a comparative capital advantage perspective may provide a useful analytical tool for studying human and intellectual mobility in broad contexts. Nevertheless, because of this article’s explorative nature, the potential to generalize findings to a broader context must be further developed. For instance, the observations made here can be further developed into hypotheses for future investigations, particularly quantitative, that compare different academic migration outcomes based on geographical attributes (e.g., comparing Asian, African, or American researchers with Europeans in China, as well as the Chinese diaspora in Europe and Chinese returnees from Europe).

Our findings also lead to a set of recommendations for researchers, academic organizations, and relevant governmental agencies. On the European side, researchers should be better informed about possible concern factors before moving to China to lower potential risks (e.g., through existing European diaspora or alumni programs in China). Furthermore, the EU and European national governments may consider that most European researchers in China would prefer to return to Europe if an opportunity arose and, thus, support new funding instruments for return migration, ease or harmonize migration regulations, and disseminate information about existing initiatives. The Chinese side should be warned that unless significant improvements are made in terms of both policies and organizational practices, European researchers working in China are unlikely to remain there. Chinese universities and research institutes should not treat foreign staff as short-term visitors but rather as long-term colleagues and allow them to retain their international exposure through research leaves or sabbaticals abroad. Additionally, Chinese employers could offer foreign staff packages that consider dual careers, healthcare, housing, and education for dependent children. These suggestions for improvement at the organizational level require changes in policy frameworks at the national level.

Finally, it remains to be seen how the COVID-19 pandemic and rapidly changing relationship between the world’s major powers will affect academic migration flows and direction of travel. Since early 2020, COVID-19 measures have effectively locked many foreigners, including academic staff, out of China, due to border closures and restrictions on in-bound travel (Lau, 2021; Mouritzen et al., 2020), which are likely to continue in the near future (Lew, 2021). Nevertheless, China will remain a scientific powerhouse, important for global knowledge production, and we can expect that research cooperation between Europe and China will continue. In a way, researchers, both European researchers in China and their Chinese counterparts in Europe, are “science diplomats” who bridge their employing institutions and lay the human foundations for the relationship between Europe and China in higher education, research, innovation, and beyond.

## Supplementary information

Below is the link to the electronic supplementary material.

Supplementary file1 (DOCX 76 KB)
