# Supplementary material for: The Experience of European Researchers in China: A Comparative Capital Advantage Perspective
Source: J Knowl Econ. 2022 Mar 7:1–29. Online ahead of print. doi: 10.1007/s13132-022-00982-3 (PMC8900960; doi:10.1007/s13132-022-00982-3)
Supplement: Supplementary file 1 — Supplementary file1 (DOCX 76 KB) [file 13132_2022_982_MOESM1_ESM.docx]

**Online Appendix: List of Interviewees**

| **Int. Nr.** | **Research Category** | **Field*** | **Priority** | <**5 or 5≥** **years in China** | **Resident City** | **Gender** | **Nationality** | **Career Stage**** | **Institution ***** |
| --- | --- | --- | --- | --- | --- | --- | --- | --- | --- |
| 1 | basic | ENS | High | <5 | Shanghai | M | Italian | Senior | 985 |
| 2 | applied | ENS | High | 5≥ | Shanghai | M | French | Senior | 211 |
| 3 | basic | ENS | Low | <5 | Beijing | F | Italian | Senior | 985 |
| 4 | basic | ENS | High | 5≥ | Shanghai | M | Swedish | Senior | RI |
| 5 | applied | SSH | Low | 5≥ | Beijing | F | Italian | Senior | 985 |
| 6 | applied | ENS | High | 5≥ | Shanghai | F | Italian | Senior | 985 |
| 7 | basic | SSH | Low | 5≥ | Shanghai | M | Spanish | Senior | 985 |
| 8 | basic | SSH | Low | 5≥ | Beijing | M | Austrian | Junior | 985 |
| 9 | applied | SSH | Low | 5≥ | Beijing | M | French | Junior | 985 |
| 10 | basic | SSH | Low | 5≥ | Beijing | F | French | Senior | 985 |
| 11 | basic | ENS | High | 5≥ | Beijing | M | Dutch | Senior | 985 |
| 12 | basic | ENS | High | <5 | Shanghai | M | French | Senior | RI |
| 13 | basic | ENS | High | 5≥ | Beijing | F | Belgian | Senior | RI |
| 14 | basic | ENS | High | 5≥ | Other | F | British | Senior | 211 |
| 15 | applied | ENS | High | <5 | Other | M | Dutch | Senior | 211 |
| 16 | applied | SSH | High | 5≥ | Shanghai | F | Finnish | Senior | 985 |
| 17 | basic | ENS | High | 5≥ | Beijing | M | Spanish | Senior | 985 |
| 18 | applied | ENS | High | <5 | Shanghai | M | Italian | Senior | 985 |
| 19 | applied | SSH | Low | 5≥ | Beijing | F | Belgian | Junior | 985 |
| 20 | basic | SSH | Low | 5≥ | Shanghai | M | French | Senior | 985 |
| 21 | applied | SSH | High | <5 | Other | M | Italian | Senior | 985 |
| 22 | applied | SSH | Low | <5 | Shanghai | M | Polish | Junior | 985 |
| 23 | applied | ENS | High | <5 | Other | M | German | Senior | RI |
| 24 | applied | SSH | Low | 5≥ | Shanghai | F | Italian | Senior | 211 |
| 25 | basic | SSH | Low | <5 | Shanghai | M | Czech | Junior | 985 |
| 26 | basic | SSH | Low | <5 | Beijing | F | Latvian | Junior | 985 |
| 27 | applied | SSH | Low | 5≥ | Beijing | M | Polish | Junior | 985 |
| 28 | basic | ENS | High | <5 | Shanghai | M | Italian | Senior | 985 |

* Engineering and natural sciences (ENS); Social sciences and humanities (SSH)

** Junior (R1, R2): R1: Early-career stage researcher (up to the point of PhD); R2: Recognised researcher (e.g., a postdoc), Senior (R3, R4): R3: Established researcher; R4: Leading researcher

*** 985: A university on the exclusive list of 39 elite institutions in Project 985

211: A university that belongs to the 112 key higher education institutions listed in Project 211

RI: Research institute
